# HIGH CONFIDENCE IN MANAGING COMMON GERIATRIC PROBLEMS AMONG RURAL FRONTLINE CLINICIANS

**DOI:** 10.1093/geroni/igae098.3852

**Published:** 2024-12-31

**Authors:** Alison Stone, Eileen Dryden, Lauren Moo, William Hung, Meaghan Kennedy, Camilla Pimentel, Jessica Riley, Thomas Caprio

**Affiliations:** Veterans Affairs, Dracut, Massachusetts, United States; Veterans Health Administration, Bedford, Massachusetts, United States; Department of Veterans Affairs, Bedford, Massachusetts, United States; James J Peters VA Medical Center, James J Peters VA Medical Center, New York, United States; Veterans Affairs, Bedford, Massachusetts, United States; VA Bedford Healthcare System, Bedford, Massachusetts, United States; Veteran’s Affairs, Bedford, Massachusetts, United States; University of Rochester Medical Center, Rochester, New York, United States

## Abstract

**Background:**

Older rural Veterans often have less access to specialty care services, including geriatrics. The Veterans Health Administration’s (VHA) GRECC Connect is a national program that provides virtual geriatric services for patients and geriatric education for rural clinicians through a case conference series focused on the 5Ms (Mind, Mobility, Medications, Multicomplexity, and Matters Most), a hallmark of high-quality geriatric care. We aimed to measure rural frontline clinicians’ confidence in managing common geriatric problems to inform future education efforts.

**Methods:**

We conducted an electronic survey-based needs assessment during three case conferences in Spring, 2024. The survey included a brief demographic section and 16 questions on respondents’ confidence addressing clinical issues within the 5Ms (e.g., polypharmacy, falls prevention, goals of care).

**Results:**

Forty nine percent (211/430) of conference participants completed the survey, including social workers, pharmacists, nurses, and physicians. On average, 69% of respondents reported being ‘confident’ or ‘very confident’ (range 53%-84%) across the 16 questions. Highest confidence levels were in the ‘Matters Most’ domain. Topics rated lower included managing depression, deprescribing, and managing behaviors in patients with dementia. Confidence varied according to health professions and the responsibilities associated with each role. Providers who serve a higher percentage of rural Veterans reported feeling more confident than their urban colleagues across all questions.

**Conclusion:**

The high level of confidence aligns with findings in the literature that report high self-efficacy ratings in the 5Ms domains among other VA providers and may be related to the VHA’s focus on Age Friendly Health Systems.

